# Assessment of Olfactory Nerve by SPECT-MRI Image with Nasal Thallium-201 Administration in Patients with Olfactory Impairments in Comparison to Healthy Volunteers

**DOI:** 10.1371/journal.pone.0057671

**Published:** 2013-02-28

**Authors:** Hideaki Shiga, Junichi Taki, Kohshin Washiyama, Junpei Yamamoto, Sakae Kinase, Koichi Okuda, Seigo Kinuya, Naoto Watanabe, Hisao Tonami, Kichiro Koshida, Ryohei Amano, Mitsuru Furukawa, Takaki Miwa

**Affiliations:** 1 Department of Otorhinolaryngology, Kanazawa Medical University, Ishikawa, Japan; 2 Department of Quantum Medical Technology, Graduate School of Medical Science, Kanazawa University, Ishikawa, Japan; 3 Department of Biotracer Medicine, Graduate School of Medical Science, Kanazawa University, Ishikawa, Japan; 4 Risk Analysis and Applications Research Group, Nuclear Safety Research Center, Japan Atomic Energy Agency, Ibaraki, Japan; 5 Department of Diagnostic and Therapeutic Radiology, Kanazawa Medical University, Ishikawa, Japan; 6 Headquarter, Kanazawa University, Ishikawa, Japan; Technical University of Dresden Medical School, Germany

## Abstract

**Purpose:**

The aim of this study was to assess whether migration of thallium-201 (^201^Tl) to the olfactory bulb were reduced in patients with olfactory impairments in comparison to healthy volunteers after nasal administration of ^201^Tl.

**Procedures:**

10 healthy volunteers and 21 patients enrolled in the study (19 males and 12 females; 26–71 years old). The causes of olfactory dysfunction in the patients were head trauma (n = 7), upper respiratory tract infection (n = 7), and chronic rhinosinusitis (n = 7). ^201^TlCl was administered unilaterally to the olfactory cleft, and SPECT-CT was conducted 24 h later. Separate MRI images were merged with the SPECT images. ^201^Tl olfactory migration was also correlated with the volume of the olfactory bulb determined from MRI images, as well as with odor recognition thresholds measured by using T&T olfactometry.

**Results:**

Nasal^ 201^Tl migration to the olfactory bulb was significantly lower in the olfactory-impaired patients than in healthy volunteers. The migration of ^201^Tl to the olfactory bulb was significantly correlated with odor recognition thresholds obtained with T&T olfactometry and correlated with the volume of the olfactory bulb determined from MRI images when all subjects were included.

**Conclusions:**

Assessment of the ^201^Tl migration to the olfactory bulb was the new method for the evaluation of the olfactory nerve connectivity in patients with impaired olfaction.

## Introduction

Many physicians find it difficult to detect malingering among olfaction-impaired patients. Current olfactory function tests (the University of Pennsylvania Smell Identification Test [1], the Connecticut Chemosensory Clinical Research Center Test [2], Sniffin sticks [3], and T&T olfactometry [4]) are useful for the analysis of olfactory function in olfaction-impaired patients. However, an abnormality in the olfactory function tests has not been used as an index for the connectivity of olfactory nerve axons passing through the cribriform plate of the ethmoid bone to enter glomeruli within the olfactory bulb in patients because it is difficult to directly view the connectivity of the peripheral olfactory nerve with current magnetic resonance imaging. On the other hand, it has previously been shown that olfactory bulb volume is correlated with odor threshold determined by using Sniffin sticks in patients with postinfectious olfactory loss [5].

The radioisotope thallium-201 (^201^Tl) migrates to the olfactory bulb after nasal administration in rodents [6], and this migration is significantly decreased by the transection of olfactory nerve fibers in mice [7]. Furthermore, The ability to detect odors and the rate of migration of ^201^Tl in the olfactory nerve are correlated in mice [8].

Nasally administered thallium-201 (^201^Tl) migrates to the olfactory bulb 24 h after ^201^Tl administration in subjects, as has been shown in healthy volunteers using a combination of single photon emission computed tomography (SPECT), X-ray computed tomography (CT), and magnetic resonance imaging (MRI) [9]. The biological safety of nasal ^201^Tl administration has also previously been shown [10].

In this study, we applied the technique of nasal administration of ^201^Tl followed by SPECT-CT and MRI imaging to patients with hyposmia due to head trauma, upper respiratory tract infection, or chronic rhinosinusitis, which are major causes of olfactory dysfunction [11], and to healthy volunteers. ^201^Tl olfactory migration in patients with impaired olfaction was compared to that in healthy volunteers. ^201^Tl olfactory migration was also correlated with the volume of the olfactory bulb determined from MRI images, as well as with odor recognition thresholds measured by using T&T olfactometry.

## Methods

### Subjects

Participants were 10 healthy volunteers (7 males and 3 females, 30–66 years old; Table 1) recruited from medical workers in the Kanazawa Medical University Hospital and Kanazawa University Hospital with normal olfactory thresholds as determined with T&T olfactometry (i.e., the odor recognition threshold score of each nostril was less than 2.0) and 21 patients with olfactory disorders receiving treatment at the smell clinic in the Kanazawa Medical University Hospital (26–71 years old; 12 males and 9 females; odor recognition threshold score: 4.2±1.4 [mean ± S.D.]; Table 1; i.e., the odor recognition threshold score of each nostril was 2.0 or more than 2.0 with self-reported olfactory disturbance). The mean age was not significantly different in the patients with head trauma, upper respiratory infection, or chronic sinusitis than in the healthy volunteers (Table 1). The numbers of smokers were not significantly different among healthy volunteer group and each patient group (Table 1).

**Table 1:** Odor recognition thresholds measured by using T&T olfactometry.

Subject Group	Age, years(Mean ± S.D.)	Gender(Male/Female)	Non-smokers/Smokersor ex-smokers	Odor recognition threshold (Mean ± S.D.)
Healthy volunteer (n = 10)	44.7±11.9	7/3	7/3	0.9±0.6
Head trauma (n = 7)	43.0±13.3	3/4	3/4	4.9±0.9*
Upper respiratory tract infection (n = 7)	50.4±10.0	4/3	6/1	3.4±1.5*
Chronic rhinosinusitis (n = 7)	54.7±15.1	5/2	4/3	4.4±1.4*

* *P*<0.05 (unpaired t-tests: comparison with healthy volunteers).

Causes of olfactory dysfunction in the patients were head trauma (n = 7), upper respiratory tract infection (n = 7), and chronic rhinosinusitis (n = 7). For head trauma subjects, types of head trauma were concussion (n = 2), cerebral contusion (n = 2), and cerebral contusion with intracranial hemorrhage (n = 3). One patient with hyposmia due to head trauma showed a shallow olfactory sulcus. Another patients with hyposmia due to head trauma showed no abnormal frontal lobe findings on MRI.

The duration of olfactory deficits at the time of the examination ranged from 4 weeks to 10 years (58 months ±53 months [mean ± S.D.]). Patients with chronic rhinosinusitis were assessed in this trial if they continued to suffer olfactory impairments even though receiving medical and/or surgical treatment. Olfactory clefts were open in all subjects.

All participants were informed of the objectives of the study and possible side effects (allergic reactions to ^201^TlCl, irritation of the digestive system, blood pressure fluctuation, or asthmatic crisis), and have given written informed consent. Subjects were excluded if they were pregnant or lactating. Exclusion criteria also included a history of kidney disease, liver injury, or other serious illness. Healthy volunteers who might have olfactory dysfunction due to head trauma, upper respiratory tract infection, or chronic rhinosinusitis were excluded in this study. Both groups of healthy volunteers (medical workers) and patients did not include nuclear medicine specialists. Therefore, their knowledge about ^201^Tl imaging may be same. The Medical Ethics Committees of Kanazawa Medical University and Kanazawa University approved this trial in advance.

### T&T Olfactometry

Each odorant was dissolved in mineral oil to form a graded series of concentrations, and then applied liberally to blotting paper and presented to subjects by using T&T olfactometry (Daiichi Yakuhin Sangyo, Tokyo, Japan). In Japan, T&T olfactometry is now a standard means of measuring olfactory thresholds. The normal odor recognition threshold score of each nostril is less than 2.0. The recognition threshold was obtained with T&T olfactometry on the side ^201^Tl nasally administered in this study.

### Merging of ^201^Tl SPECT Images with MRI Images and Analysis of Olfactory Bulb Volume

All procedures involving the handling of radioisotopic materials were performed in the Division of Radiology at Kanazawa Medical University or the Division of Radioisotopes at Kanazawa University Hospital. A solution of ^201^TlCl in saline (74 MBq/mL) was obtained from Nihon Medi-Physics (Tokyo, Japan). For each subject, 0.3 mL ^201^TlCl saline solution (22 MBq) was instilled via syringe into the olfactory cleft in the right or left larger nasal cavity. The unilateral ^201^Tl nasal administration was performed in this study, because uptake of ^201^Tl in the right or left distinction may be unclear in the case of bilateral ^201^Tl nasal administration. Under endoscopic findings, we choose the side of larger olfactory cleft to be assessed for the certain nasal administration to the olfactory cleft. After ^201^Tl nasal administration, subjects laid on their sides for 30 min. Uptake of ^201^Tl was assessed from SPECT scans performed 24 h after ^201^Tl administration. It has been shown that ^201^Tl migration from the olfactory epithelium to the olfactory bulb is visible 24 h after ^201^Tl nasal administration in healthy volunteers [9]. Each SPECT scan was 30 min in duration and was followed by an X-ray CT scan to localize the nasal cavity and olfactory bulb. The data acquisition was started using a dual-headed SPECT-CT hybrid system (Symbia T6; Siemens Japan Healthcare, Japan) equipped with low-energy, high-resolution collimators. Data were acquired over 360 degrees, with 90 projections (30 s per projection in continuous rotation), 128 × 128 matrix (with 2.29-times zoom, pixel size 2.1 mm), and 72 KeV photopeak with a 30% main window and 7% subwindow on both sides. Data reconstruction underwent by using ordered subsets expectation maximization (9 subsets and 8 iterations) with 3-dimensional resolution recovery, CT-based attenuation correction, and scatter correction [12], [13] (Flash 3D, Siemens Japan Healthcare, Japan).

For each subject, MRI T2 weighted images (Syngo MR B17 3.0T; Siemens Japan Healthcare, Japan, or Signa HDx 3.0T; GE Healthcare-Japan, Japan) were collected separately from the SPECT scan, one day before. Each MRI image was merged with the corresponding SPECT image through fusion with the CT part of the SPECT-CT by using the mutual information–based registration method [14]. Experienced radiologists, (N.W. and H.T.) who were blind to the olfactory test data, separately determined the volume of the olfactory bulb by manual segmentation of MRI coronal slices (2-mm slice thickness) through the olfactory bulb on the side of nasal administration. The change of diameter at the beginning of the olfactory tract was used as the proximal demarcation of the olfactory bulb. The average scores of those were used for the statistical analysis. Olfactory bulb volume was not regarded as criteria for the selection of patients.

### Analysis of ^201^Tl Counts on the SPECT-MRI Image

Two regions of interest for the nasal turbinate area and the anterior skull base (olfactory bulb area) were set on the ^201^Tl SPECT–MRI fusion image. On 3 sequential fused images in both sagittal and coronal planes, large regions of interest were tentatively set manually on the nasal turbinate area to cover all of the residual ^201^Tl activity in the nasal cavity. The nasal cavity region of interest was defined as the area bounded by the 50% threshold of the peak ^201^Tl count and delineated. Then, oval regions of interest were set manually to delineate the olfactory bulb on the side of nasal administration of the tracer by referencing the MRI T2 weighted images. The size of the oval olfactory bulb region of interest was approximately 8 to 9 pixels on the long axis and 3 to 4 pixels on the short axis in the sagittal image and 5 to 6 pixels by 2 to 3 pixels in the coronal image. The regions of interest were set excluding the sphenoidal sinus area and the nasopharyngeal area.

The index of ^201^Tl migration from the olfactory epithelium to the olfactory bulb was determined as the ratio of the total ^201^Tl counts in the olfactory bulb region of interest to the total ^201^Tl counts in the nasal turbinate region of interest, expressed as a mean percentage of the values calculated from both sagittal and coronal images. Experienced nuclear radiologists (J.T. and K.O.) who were blind to the olfactory test data determined separately the two regions of interest on the SPECT-MRI image. The average scores of those were used for the statistical analysis.

### Phantom Study

A phantom study was performed with the same data acquisition and reconstruction method used in the clinical study. In a cylindrical phantom (20 cm in diameter) filled with water, a ^201^Tl (40 µCi) spherical source (4 mm in diameter) was positioned on the center of the rotation. Then the counts of pixels on the line passing through the center of the source and at a right angle to the axis of camera rotation were measured. Full width at half maximum (FWHM) was 7.0 mm. When normalized by the maximum pixel count (7972 counts) of the spherical source, pixel counts at 1 (2.1 mm), 2 (4.2 mm), 3 (6.3 mm), 4 (8.4 mm), and 5 (10.5 mm) pixels from the center of the source were 0.76, 0.34, 0.09, 0.016, 0.001, respectively. Thus, when the olfactory bulb is located more than 4 pixels (8.4 mm) from the high radioactivity in the nasal cavity, the count contamination to the olfactory bulb will be less than 2% of the activity of the nasal cavity.

### Statistical Analysis

Two-tailed Spearman correlations, unpaired t-tests, Bonferroni’s multiple comparison test, and Kruskal-Wallis tests were performed using Prism 5 software (GraphPad, San Diego, CA, USA). *P* values less than 0.05 were considered significant. A sample size of 31 subjects gave 99.9% power (alpha = .05, two-tailed) for comparing nasal ^201^Tl migration to the olfactory bulb in 4 groups (JMP software, Cary, NC, USA).

## Results

### Reductions in Nasal ^201^Tl Migration to the Olfactory Bulb in Patients

**Figure 1:**
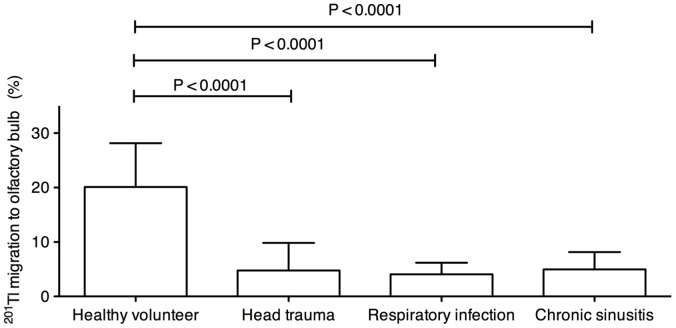
^201^Tl migration in patients with impaired olfaction in comparison to healthy volunteers. Nasal ^201^Tl migration to the olfactory bulb in healthy volunteers (n = 10) and patients with impaired olfaction due to head trauma (n = 7), upper respiratory tract infection (respiratory infection; n = 7), or chronic rhinosinusitis (n = 7). *P* values were obtained with Bonferroni’s multiple comparison test and the Kruskal-Wallis test. Bars: Mean ± S.D.

To determine whether the viability or function of the peripheral olfactory nerve was reduced in the patients with impaired olfaction, we assessed migration of nasally administered ^201^Tl to the olfactory bulb in the patients and healthy volunteers. Migration of nasal ^201^Tl to the olfactory bulb was significantly lower in the patients with head trauma, upper respiratory infection, or chronic sinusitis than in the healthy volunteers (Fig. 1; Kruskal-Wallis test for comparing 4 groups, *P* = 0.0004; unpaired t-tests for comparing 2 groups: head trauma, *P* = 0.0005; upper respiratory tract infection, *P* = 0.0001; chronic sinusitis, *P* = 0.0003; Bonferroni’s multiple comparison test for comparing 2 groups: head trauma, *P*<0.0001; upper respiratory tract infection, *P*<0.0001; chronic sinusitis, *P*<0.0001). There were no significant differences between each patient group in migration of nasally administered ^201^Tl to the olfactory bulb.

**Figure 2:**
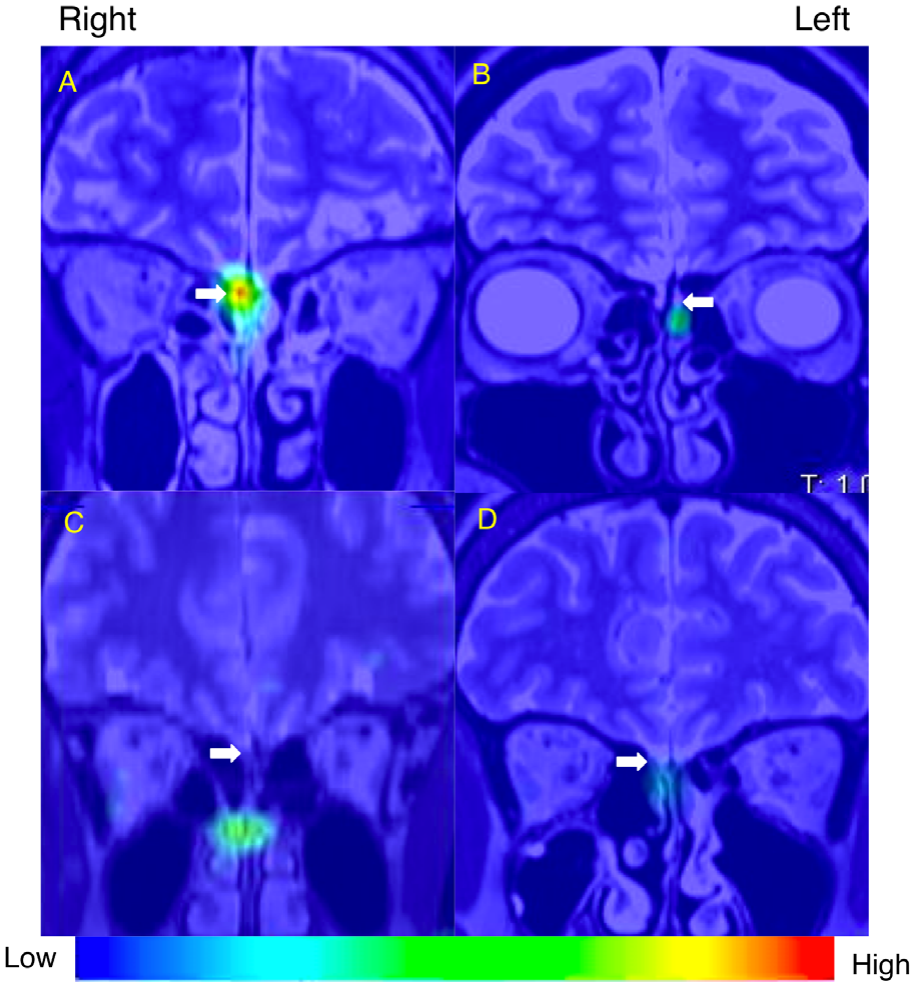
SPECT-MRI fusion image (coronal view) of nasal ^201^Tl migration to the olfactory bulb. White arrows indicate the olfactory bulb and olfactory nerve. (A) A 60-year-old healthy male volunteer. (B) A 44-year-old female with hyposmia after head trauma. (C) A 42-year-old female with hyposmia after upper respiratory tract infection. (D) A 67-year-old female with hyposmia due to chronic rhinosinusitis. The index of ^201^Tl migration from the olfactory epithelium to the olfactory bulb in the selected subjects was shown in Table 2.

**Figure 3:**
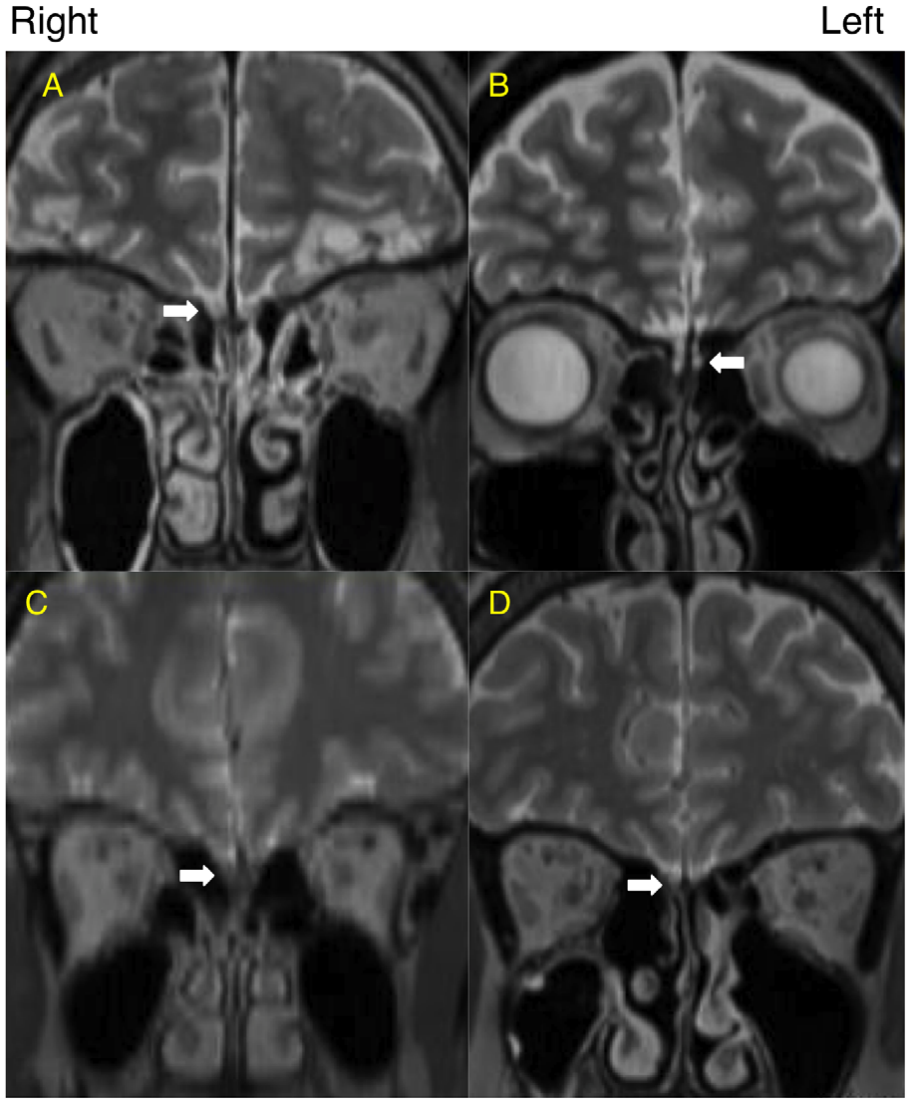
MRI image (T2 weighted, coronal view). White arrows indicate the olfactory bulb and olfactory nerve. (A) A 60-year-old healthy male volunteer. (B) A 44-year-old female with hyposmia after head trauma. (C) A 42-year-old female with hyposmia after upper respiratory tract infection. (D) A 67-year-old female with hyposmia due to chronic rhinosinusitis.

Representative cases are shown in Fig. 2 and summarized in Table 2. A 60-year-old healthy male volunteer showed good olfactory function and a high level of nasal ^201^Tl migration to the olfactory bulb (Fig. 2A, Table 2), a 44-year-old female with hyposmia after head trauma showed severe olfactory dysfunction and a low level of nasal ^201^Tl migration to the olfactory bulb (Fig. 2B, Table 2), a 42-year-old female with hyposmia after upper respiratory tract infection showed moderate olfactory dysfunction and a low level of nasal ^201^Tl migration to the olfactory bulb (Fig. 2C, Table 2), and a 67-year-old female with hyposmia from chronic rhinosinusitis showed moderate olfactory dysfunction and a low level of nasal ^201^Tl migration to the olfactory bulb (Fig. 2D, Table 2). MRI images of the representative cases are shown in Fig. 3.

**Table 2:** Functional olfactory measurements in selected subjects.

Selected subjects	Nasal ^201^Tl migration to the olfactory bulb, %	Odor recognition threshold
60-year-old healthy male volunteer	29.0	1.4
44-year-old female with hyposmia after head trauma	4.2	5.8
42-year-old female with hyposmia after upper respiratory tract infection	4.5	4.8
67-year-old female with hyposmia due to chronic rhinosinusitis	5.0	3.2

### Nasal ^201^Tl Migration to the Olfactory Bulb and Olfactory Function Assessed by T&T Olfactometry

To assess whether nasal ^201^Tl migration to the olfactory bulb reflects olfactory function, we examined the relationships between the recognition threshold obtained with T&T olfactometry and the amount of nasal ^201^Tl that migrated to the olfactory bulb in the subjects.

**Figure 4:**
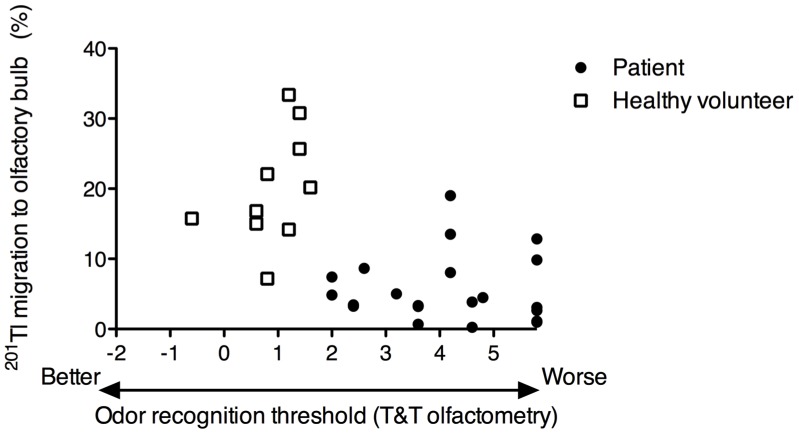
Correlation between olfactory ^201^Tl migration and olfactory function as assessed by T&T olfactometry. Nasal ^201^Tl migration to the olfactory bulb as a function of odor recognition threshold in patients and healthy volunteers (n = 31). Spearman *r* = –0.62, *P* = 0.0002. Closed circles, patients; Open squares, healthy volunteers.

Nasal ^201^Tl migration to the olfactory bulb was correlated with the odor recognition threshold obtained by T&T olfactometry (Spearman *r* = –0.62, *P* = 0.0002) in the healthy volunteers and the olfaction-impaired patients evaluated as a single group (Fig. 4). Nasal ^201^Tl migration to the olfactory bulb was not correlated with duration of olfactory deficits at the time of the examination in the patients with impaired olfaction (Spearman *r* = 0.33, *P* = 0.15).

### Nasal ^201^Tl Migration to the Olfactory Bulb and Olfactory Bulb Volume Determined from MRI Images

To assess whether nasal ^201^Tl migration to the olfactory bulb reflects olfactory bulb size, we examined the relationships between the olfactory bulb volume determined from MRI images and the amount of nasal ^201^Tl that migrated to the olfactory bulb in the subjects. Olfactory bulb volume was significantly lower in the patients with head trauma, upper respiratory infection, or chronic sinusitis than in the healthy volunteers (Table 3).

**Table 3:** Olfactory bulb volume measurements in subjects.

Subject Group	Olfactory bulb volume, mm^3^ (Mean ± S.D.)
Healthy volunteer (n = 10)	83.2±25.5
Head trauma (n = 7)	22.4±7.5*
Upper respiratory tract infection (n = 7)	35.8±12.3*
Chronic rhinosinusitis (n = 7)	33.3±10.5*

* *P*<0.05 (unpaired t-tests: comparison with healthy volunteers).

**Figure 5:**
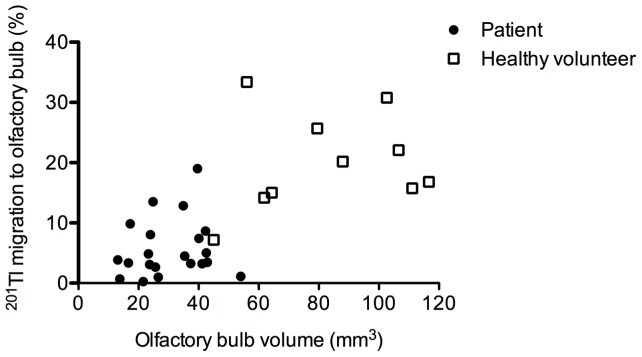
Correlation between olfactory ^201^Tl migration and unilateral olfactory bulb volume as determined by using MRI images. Nasal ^201^Tl migration to the olfactory bulb as a volume of olfactory bulb in patients and healthy volunteers (n = 31). Spearman *r* = 0.73, *P*<0.0001. Closed circles, patients; Open squares, healthy volunteers.

Nasal ^201^Tl migration to the olfactory bulb was correlated with the olfactory bulb volume determined from MRI images (Spearman *r* = 0.73, *P*<0.0001) in the healthy volunteers and the olfaction-impaired patients evaluated as a single group (Fig. 5).

## Discussion

Nasal ^201^Tl migration to the olfactory bulb was reduced in the patients with impaired olfaction due to head trauma, upper respiratory tract infection, and chronic rhinosinusitis, which are major causes of olfactory dysfunction [11] relative to the values in healthy volunteers. The assessment of olfactory nerve damage using our imaging method may be useful for scanning the lesion of the olfactory nerve connectivity in patients with impaired olfaction. Patients with impaired olfaction due to major causes of olfactory dysfunction would have damage of olfactory mucosa, because histological changes have been reported in the olfactory mucosa of patients with olfactory deficits due to head trauma [15], [16], upper respiratory tract infection [17], or chronic sinusitis [18]. The degree of axon degeneration in human olfactory mucosa correlates with olfactory function [19]. Decreased peripheral olfactory neuron in olfactory mucosa of the patients with impaired olfaction may reduce the nasal ^201^Tl migration to the olfactory bulb.

The migration of ^201^Tl to the olfactory bulb was correlated with odor recognition thresholds obtained with T&T olfactometry when all subjects were included. Furthermore, the volume of the olfactory bulb determined by using MR images was positively correlated with the level of^ 201^Tl migration from the nasal turbinate area to the olfactory bulb on SPECT-MRI fusion images in both the healthy volunteers and patients with impaired olfaction. Our results suggest that the connectivity of olfactory neurons between the olfactory bulb and olfactory mucosa reflect the volume of the olfactory bulb. Increasing olfactory stimulation leads to decreased cell mortality in the olfactory bulb *in vivo* [20]. Reduced connectivity of olfactory neurons between the olfactory bulb and olfactory mucosa may effect on olfactory bulb volume with a decrease of olfactory stimulation in the patients with impaired olfaction.

The nasal ^201^Tl migration to the olfactory bulb was not significantly correlated with olfactory bulb volume determined by using MR images in the healthy volunteers or in the patients with impaired olfaction evaluated as separate groups (data not shown), likely because of the small number of subjects in each group. It has been shown that olfactory bulb volume is larger in men than in women [21]. It warrants further investigation whether gender would effect on the nasal ^201^Tl migration to the olfactory bulb.

In this study, the decrease of the migration of ^201^Tl to the olfactory bulb was not significantly different among the patients with head trauma, upper respiratory tract infection, or chronic sinusitis. The degree of peripheral olfactory nerve degeneration may be similar in the patient groups included in this study, because we observed no significant different olfactory thresholds among the patient groups.

In this study, we observed no adverse effects in the subjects. Unlike intravenous administration of radiopharmaceutical agents, which would deliver only small amounts of radiation to the nasal cavity, nasal administration of ^201^Tl delivers a high radiation dose to the nasal cavity. Therefore, the estimation of the absorbed dose in the nasal cavity and neighboring organ such as brain and lens are important. We calculated the absorbed dose of ^201^Tl in the nasal cavity in a previous clinical study, and its dose was too low for acute radiation effects [9]. The brain and eyes are separated from the nasal cavity by the nasal bone, ethmoturbinals, and the sphenoidal bone. A Monte Carlo simulation using the ICRP reference adult male [22] showed that almost all conversion electrons and Auger electrons emitted from ^201^Tl in the nasal cavity were stopped in the nasal cavity and were not absorbed by neighboring organs (data not shown). Therefore, gamma-rays and X-rays from the ^201^Tl in the nasal cavity contributed the doses of ^201^Tl absorbed in the brain and lens. Our preliminary calculation showed that the absorbed doses in the brain and lens after nasal administration of 22 MBq ^201^Tl were 0.067 mGy and 0.59 mGy, respectively. Therefore, acute radiation effects in the brain and lens can be also avoided.

The majority of ^201^Tl administered intranasally in this study migrated to the nasopharyngeal region and then was swallowed. It has been shown that intravenously administered ^201^Tl is hardly absorbed by the central nervous system, including the olfactory bulb, *in vivo* [6]. Therefore, the potential systemic effects of swallowed ^201^Tl can be ignored. Residual activity in the olfactory epithelium may be independent from mucosal clearance of the administered ^201^Tl, because mature olfactory neurons have immotile cilia [23].

It will be important to study whether an increase in ^201^Tl migration to the olfactory bulb during treatment is correlated with a decrease in odor recognition thresholds in patients with olfactory disorders. The recovery rate of olfactory function in patients with traumatic olfactory dysfunction is less than 30% [24]. In this study, we did not assess follow-up data in the subjects, but ^201^Tl migration can be used to visualize olfactory nerve regeneration *in vivo* [25]. Our imaging method could be especially helpful for assessing the connectivity of the peripheral olfactory nerve in patients with olfactory disorders during treatment and predicting the course of olfactory impairment. Patients with intact olfactory nerve fibers may be well selected by means of a new isotope imaging technique for the long-term treatment of olfactory dysfunction.

### Conclusions

Nasal ^201^Tl migration to the olfactory bulb was reduced in patients with impaired olfaction due to head trauma, upper respiratory tract infection, and chronic rhinosinusitis, which are major causes of olfactory dysfunction when compared to the ^201^Tl migration in healthy volunteers. Assessment of the ^201^Tl migration to the olfactory bulb was the new method for the evaluation of the olfactory nerve connectivity in patients with olfactory disorders.
